# Elucidation of Plasma-induced Chemical Modifications on Glutathione and Glutathione Disulphide

**DOI:** 10.1038/s41598-017-13041-8

**Published:** 2017-10-23

**Authors:** Christina Klinkhammer, Christof Verlackt, Dariusz śmiłowicz, Friederike Kogelheide, Annemie Bogaerts, Nils Metzler-Nolte, Katharina Stapelmann, Martina Havenith, Jan-Wilm Lackmann

**Affiliations:** 1 Physical Chemistry II, Ruhr University Bochum, 44780 Bochum, Germany; 2 PLASMANT, University of Antwerp, 2610 Wilrijk, Antwerp, Belgium; 3 Inorganic Chemistry I—Bioinorganic Chemistry, Ruhr University Bochum, 44780, Bochum, Germany; 4 Biomedical Applications of Plasma Technology, Ruhr University Bochum, 44780 Bochum, Germany; 5 Plasma for Life Sciences, Department of Nuclear Engineering, North Carolina State University, Raleigh, NC, 27695 USA; 6 ZIK plasmatis at Leibniz Institute for Plasma Science and Technology e.V. (INP Greifswald e.V.), Felix-Hausdorff-Str. 2, 17489 Greifswald, Greifswald, Germany

## Abstract

Cold atmospheric pressure plasmas are gaining increased interest in the medical sector and clinical trials to treat skin diseases are underway. Plasmas are capable of producing several reactive oxygen and nitrogen species (RONS). However, there are open questions how plasma-generated RONS interact on a molecular level in a biological environment, e.g. cells or cell components. The redox pair glutathione (GSH) and glutathione disulphide (GSSG) forms the most important redox buffer in organisms responsible for detoxification of intracellular reactive species. We apply Raman spectroscopy, mass spectrometry, and molecular dynamics simulations to identify the time-dependent chemical modifications on GSH and GSSG that are caused by dielectric barrier discharge under ambient conditions. We find GSSG, S-oxidised glutathione species, and S-nitrosoglutathione as oxidation products with the latter two being the final products, while glutathione sulphenic acid, glutathione sulphinic acid, and GSSG are rather reaction intermediates. Experiments using stabilized pH conditions revealed the same main oxidation products as were found in unbuffered solution, indicating that the dominant oxidative or nitrosative reactions are not influenced by acidic pH. For more complex systems these results indicate that too long treatment times can cause difficult-to-handle modifications to the cellular redox buffer which can impair proper cellular function.

## Introduction

Cold atmospheric pressure plasmas are gaining increasing interest in clinical trials for improved healing of chronic wounds^1^ and several other skin-related diseases. Typical plasma sources for wound healing can be categorized in direct plasma sources, *e.g*. dielectric barrier discharges (DBDs)^2^, or indirect plasma sources, *e.g*. jets^3^. While the direct treatment allows contact with the active plasma zone, indirect treatment typically means contact of the treated object with an effluent of the plasma, thus with only long-living species^4^. DBDs are often generated directly in ambient air, relinquishing the need for additional feed gas systems and allow for a more uniform treatment of larger surfaces. The presence of both nitrogen and oxygen under ambient conditions allows for all manner of reactive oxygen and nitrogen species (RONS) to be generated, which in turn can interact with the area of interest. Several studies have been published where the various reactive species have either been measured^5–7^ or simulated^8–10^ when the discharge is in contact with a water-containing surface. While results are promising, interactions of eukaryotic cells and cellular components with plasma are still under investigation. Several groups investigated the impact of plasma treatment on various cells and typical results include the induction of DNA damage^11,12^, cell membrane damage^13^, and oxidative stress^14^ (for a review of bactericidal mechanisms, see^15^). While moderate treatment typically results in non-permanent damage^16^, longer treatment times can induce, *e.g*., apoptosis^17^. One investigated topic is the impact on glutathione (GSH) and glutathione disulphide (GSSG)^18–20^. These molecules form the cellular redox buffer system including various enzymes correlated to GSH to GSSG conversion and recycling. Two molecules of GSH can easily be converted into GSSG by forming a disulphide bond either in the presence of reactive species or even faster by enzymatic pathways^21,22^. Glutathione peroxidases, as well as other enzymes, rapidly convert two GSH to GSSG, while reducing and thereby detoxifying various hydroperoxides. In reverse, several enzymes, such as glutathione reductase, catalyse the recycling of GSSG back to two GSH^22^. This recycling mechanism allows for an efficient detoxification of intracellular reactive species, which might otherwise cause uncontrolled and undesired modifications at other biomolecules. Besides the formation of GSSG, GSH can also be chemically modified by other electrophiles and is finally removed from the cell. Under certain conditions, twice or thrice-fold oxidation of the thiol group of GSH, resulting in sulphinic or sulphonic acid (GSO_2_H and GSO_3_H, respectively), pose a problem for the cellular recycling mechanisms. While some studies report GSO_2_H to be still reversible by cellular means, GSO_3_H is always described as irreversible^23^. Several groups already demonstrated that treatment with cold atmospheric plasma exerts a strong impact on sulphur-carrying amino acids^24^ and can also cause the intracellular GSH level to drop in a matter of seconds. Ishaq *et al*. described a reduction of intracellular GSH levels in melanoma cells treated with a helium-fed plasma jet by 80% after 30 s of exposure^25^. In addition, Zhao and colleagues demonstrated a strong decrease in free GSH using a helium-oxygen plasma jet system to treat hepatoma cells^26^. Furthermore, Ke *et al*. showed that after treatment with a pin-to-plate system in argon atmosphere for as little as two minutes, disulphide bonds are formed, indicating the conversion of GSH to GSSG^20^.

Here, we demonstrate that a DBD already used in clinical trails^27,28^ also affects GSH levels. To further understand how plasma impacts on GSH and what impact plasma treatment can have on the cellular redox system in general, experimental results from Raman spectroscopy and mass spectrometry (MS) measurements are combined with molecular dynamics (MD) simulations to elucidate chemical modifications caused on both, GSH and GSSG.

## Results

### Ellman’s assay

In a first step, the time-dependent impact of DBD treatment on the thiol group of GSH molecules was investigated. While several groups^26,29^ have already observed the loss of free thiols after plasma treatment, no such studies were performed with this specific source. Ellman’s assay data showed that free thiols are lost in the sample in a time-dependent manner. Treatment with the DBD for 1 min already caused a significant loss of free thiols. When extending treatment times to 5 min, only minor amounts of free thiols, compared to untreated samples, could be observed, indicating that most thiol moieties could be expected to be modified after 5 min of DBD treatment. Complete loss of free thiols could be observed after 20 min of treatment.

### Raman spectroscopy

**Figure 1:**
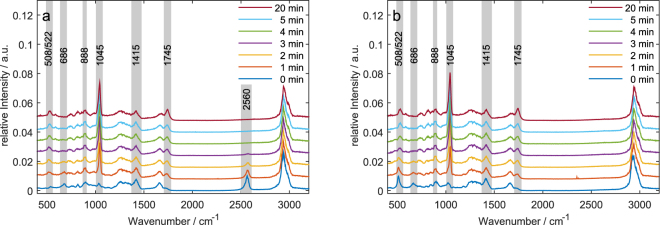
Raman spectra of plasma treated GSH (**a**) and GSSG (**b**) in distilled water. Bands which are discussed are highlighted in grey.

In order to identify the chemical modifications of GSH and GSSG induced by plasma treatment, we investigated the samples by Raman spectroscopy (Fig. 1). We monitored changes depending on the treatment time for several spectral features. The vibrational bands and their assignment are listed in Table 1.

**Table 1:** Assignment of peaks of GSH an GSSG found in the Raman spectra.

Wavenumber /cm^−1^	Assignment	References
2950	C-H stretching of CH_2/3_	34
2560	S-H stretching	35
1745	-COO-H stretching	36
1660	Amide I	34
1415	COO^-^ symmetric stretching	36
1200–1370	Amide III	34
1045	cysteic acid, S = O stretching	37
888	S-N = O bending	33
820	-C(O)-OH stretching	35
686	C-S stretching	35
522	S-N stretching	33
508	S-S stretching	37

For GSH, we observed a decrease in intensity of the band at 2560 cm^−1^ that was assigned to the S-H stretching mode (ν(S-H)). A similar change of GSH has also been observed for other plasma set-ups^20,30^ as well as for cysteine and other cysteine containing molecules^31,32^ upon DBD treatment. In accordance with these studies, we assigned this to the oxidation of the sulphur moiety. Compared to the studies of Ke and Ma^20,30^, we also observed the formation of GSSG indicated by the rise of the band at 508 cm^−1^, which can be annotated to the stretching motion of the disulphide bond (ν(S-S)). However, we observed additional other oxidation products besides GSSG. The most prominent change was the rise of a sharp peak at 1045 cm^−1^ that was assigned to the symmetric S = O stretching mode (ν(S = O)). This has also been observed before for cysteine and cysteine-containing molecules upon DBD treatment^31,32^. Here, the observation of the stretching mode ν(S = O) can be attributed to the formation of glutathione sulphonic acid (GSO_3_H) analogously to the oxidation of cysteine to cysteic acid^31^. Furthermore, the spectrum showed an increase of the band at 1745 cm^−1^, whereas the intensity of the band at 1415 cm^−1^ decreased. The former could be assigned to the stretching mode of -COO-H (ν (COO-H)) while the latter was assigned to the symmetric stretching of COO^-^ (ν (COO^-^)). In a second step we investigated the modifications of GSSG under plasma treatment itself. Here, the increase of the ν(S = O) peak at 1045 cm^−1^ due to the oxidation of sulphur was also observed. Further, these spectra displayed a strong band in a spectral range of 500–530 cm^−1^ that was assigned to two partially overlapping bands: The ν(S-S) of GSSG centred at 508 cm^−1^ and ν(S-N) of GSNO at 522 cm^−1^. Interestingly, DBD-treated GSH and GSSG samples showed a similar response in terms of prominent Raman bands: While GSH is converted to GSSG during short treatment times, GSSG appears only as an intermediate product during plasma-induced modification processes. In both cases, GSO_3_H and S-nitrosoglutathione (GSNO) seem to be the dominant end products after 5 min of treatment. The latter assignment was supported by the additional presence of the S-N = O bending mode (δ (S-N = O)) at 888 cm^−1^. Both peaks have been reported in Raman spectroscopic studies of GSNO^33^. For increasing plasma treatment times, we observed a change of this convoluted band due to a change in relative intensity of the two overlapping bands at 508 cm^−1^ and 522 cm^−1^ caused by a change in chemical composition The contribution of GSSG is found to decrease while the contribution of GSNO increased which explains the observed change of the total intensity at in the spectral range of 500–530 cm^−1^. The same band was also found for GSH, while this band did not show these intensity changes.

**Figure 2:**
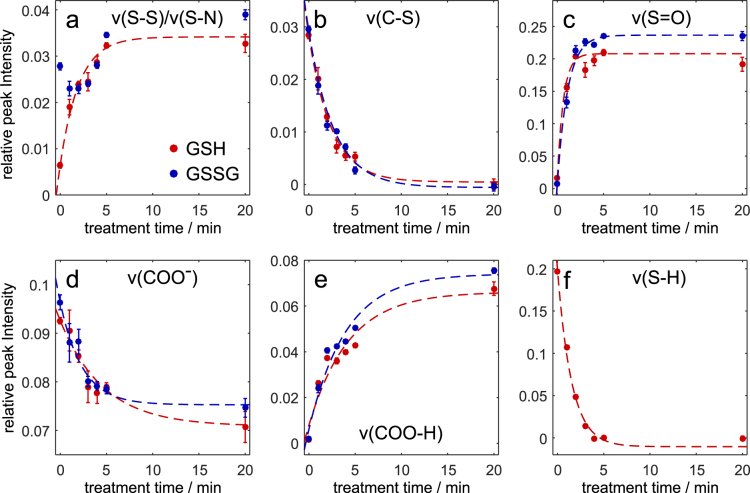
Integrated intensities for the following bands of GSH (orange) and GSSG (blue) in distilled water. Exponential trends are included as guide to the eye. (**a**) ν(S-S)/ν(S-N), 508/522 cm^−1^; (**b**) ν (C-S), 686 cm^−1^; (**c**) ν(S = O), 1045 cm^−1^; (**d**) ν (COO^-^), 1415 cm^−1^; (**e**) ν (COO-H), 1745cm^−1^; (**f**) ν(S-H), 2560 cm^−1^. Error bars indicate 95% confidence interval.

Furthermore, the spectra showed other changes that indicated further oxidation products. Additionally, the peak at 686 cm^−1^ originating from the C-S stretching mode (ν (C-S)) disappeared gradually with longer treatment times, which indicated a cleavage of the C-S bond. In Fig. 2, the integrated band intensities were plotted with respect to the plasma treatment time.

In most cases, the peaks showed a rather exponential trend for the change in intensity (decrease or increase) with exception of the combined peak of ν(S-S) and ν(S-N) in the spectra of GSSG. Here, we observed an initial decrease between 0 min and 1 min followed by an increase. This points towards a rapid cleavage of the disulphide bond and the successive formation of GSNO.

**Figure 3:**
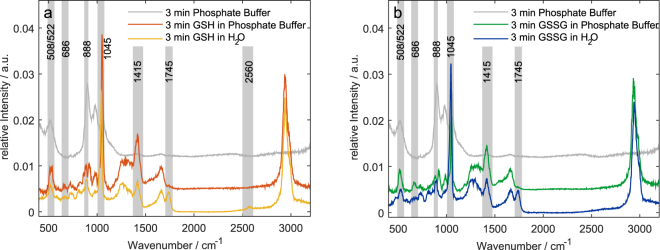
Comparison of the pure sodium phosphate buffer, buffered and unbuffered GSH (**a**) and GSSG (**b**) after 3 min treatment time. The peaks of interest are highlighted in grey.

Taking into account the strong changes in the ν (COO-H) and ν (COO-) (Fig. 2d and e), it became apparent that a reprotonation of the carboxyl group occurred^36^ in a plasma exposure-dependent manner. pH measurements resulted in a decrease of pH down to pH $\approx$ 1 after plasma treatment times of 5 min or longer. To investigate if the decrease in pH affects the formation of oxidative and nitrosative modifications at the sulphur moiety, the same experiments were repeated in sodium phosphate buffer at approximately neutral pH. In order to remove the strong phosphate signals originating from the buffer, the Raman spectra had to be further processed by subtracting the phosphate spectrum before the sample signals could be analysed. As a result, several peaks in these difference spectra were less apparent than in the spectra of the unbuffered samples. The spectra of the pure buffer, buffered and unbuffered samples after 3 min treatment time are compared in Fig. 3. We have to point out, that the peaks of ν(S-S), δ (S-N = O) and ν(S = O) are overlapping with buffer signals.

**Figure 4:**
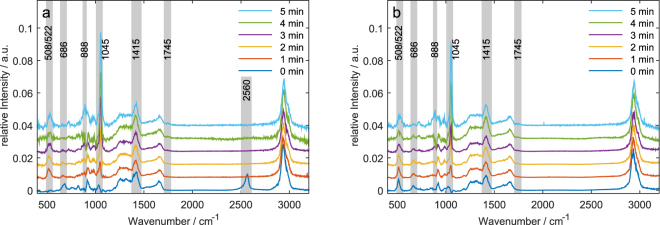
Raman spectra of GSH (**a**) and GSSG (**b**) in sodium phosphate buffer after plasma treatment of 0–5 min. Here, we show the difference spectra obtained by subtraction of pure plasma treated phosphate buffer from background corrected sample spectra each normalized to a phosphate peak at 400 cm^−1^. The peaks discussed in the text are highlighted in grey.

Despite the overlap with a phosphate band, several features could clearly be identified (Fig. 4). The ν(S = O) peak was clearly visible and showed an increase in intensity upon plasma exposure. The smaller peaks for ν(S-S) and ν (C-S) were still visible, however a clear trend could not be seen due to the overlap with phosphate bands: *E.g*., the δ (S-N = O) mode could hardly be observed in the difference spectra. As for GSH and GSSG in pure water, the amide I band at 1660 cm^−1^ and amide III band at 1200–1370 cm^−1^ did not change significantly with longer treatment times. In contrast to treatment in water, we did not find any peak at the ν (COO-H) band position at 1745cm^−1^, while the ν (COO-) peak remained constant in intensity, indicating that while the protonation state of the carboxyl group stays relatively constant, oxidative and nitrosative modifications occurred in the same way as with unbuffered solution.

A second striking difference for GSH was the complete disappearance of ν(S-H) at 2560 cm^−1^ after 1 min of treatment time. Due to the overlap with a phosphate band, any analysis of the ν(S-S) peak at 508 cm^−1^ and the SNO peaks at 522 cm^−1^ and 888 cm^−1^ was rather challenging. In summary, we conclude qualitatively, that similar as for unbuffered glutathione GSNO is produced, however no quantitative estimate can be provided.

### Mass spectrometry

**Figure 5:**
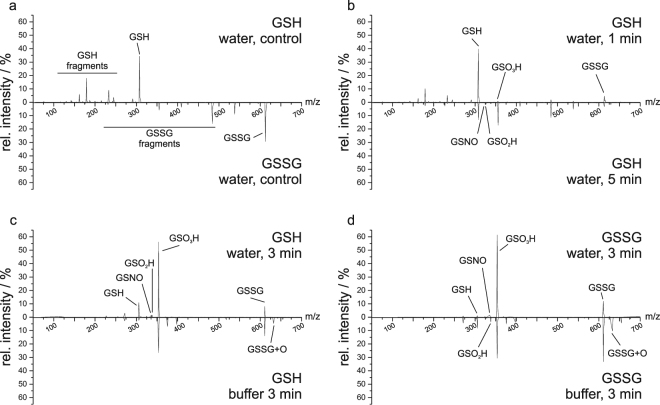
Mirror plots of MS survey scans. Mirror plots are chosen to show controls (**a**), the influence of treatment time on GSH in water (**b**), and to compare the relevance of pH-buffered medium on the resulting chemical modifications at GSH (**c**) and GSSG (**d**). (**a**) and (**b**) were measured in positive mode, (**c**) and (**d**) in negative mode to reduce the buffer influence. The intensities were calculated showing the intensity percentage of a peak relative to the spectrum’s total intensity, to alleviate the significant lower counts in negative mode; peaks are annotated with the molecule of corresponding mass; “buffer” indicates the presence of 0.15 M sodium phosphate buffer set to pH 7. GSH and GSSG fragments indicate the presence of in-source fragments of the corresponding molecule.

To clarify and support the Raman analysis, we performed MS of plasma-treated GSH and GSSG (Fig. 5). Samples were measured using both positive and negative ionization modes to cover as many modified species as possible and to mitigate contamination of mass spectra by buffer ions. Samples treated both in distilled water or in sodium phosphate buffer were analysed to investigate how pH conditions impact on the generation of modified target molecules. The MS data were in good agreement with the Raman data in several ways. While the loss of GSH during treatment was expected, it could also be observed that the intensity of the GSSG peak first increased during treatment of GSH. From 5 min of treatment and onwards it started to decrease, indicating the loss of previously formed GSSG. After DBD treatment, GSO_3_H could be observed as an increasingly dominant signal, indicating that oxidation occurs mainly at the thiol moiety of GSH. For short treatment times, the rise of a peak assigned to GSO_2_H was found to eventually decrease again. Interestingly, treatment of GSSG resulted in the loss of GSSG signal and increase of oxidised GSH signals, such as GSO_3_H, as well. Furthermore, GSNO could also be observed after treatment, though intensities were rather low, which might be due to suboptimal ionization efficacy compared to GSO_3_H. Nevertheless, signal intensities of GSNO were relatively stable even after longer treatment times. In addition, comparing samples in buffered versus non-buffered liquid, it was evident that while the general oxidative and nitrosative modifications induced by DBD treatment were identical, the relative peak intensities were affected by treatment under stable pH conditions, which could also be observed in the Raman data.

### Reactive MD simulations using the DFTB method

**Figure 6:**
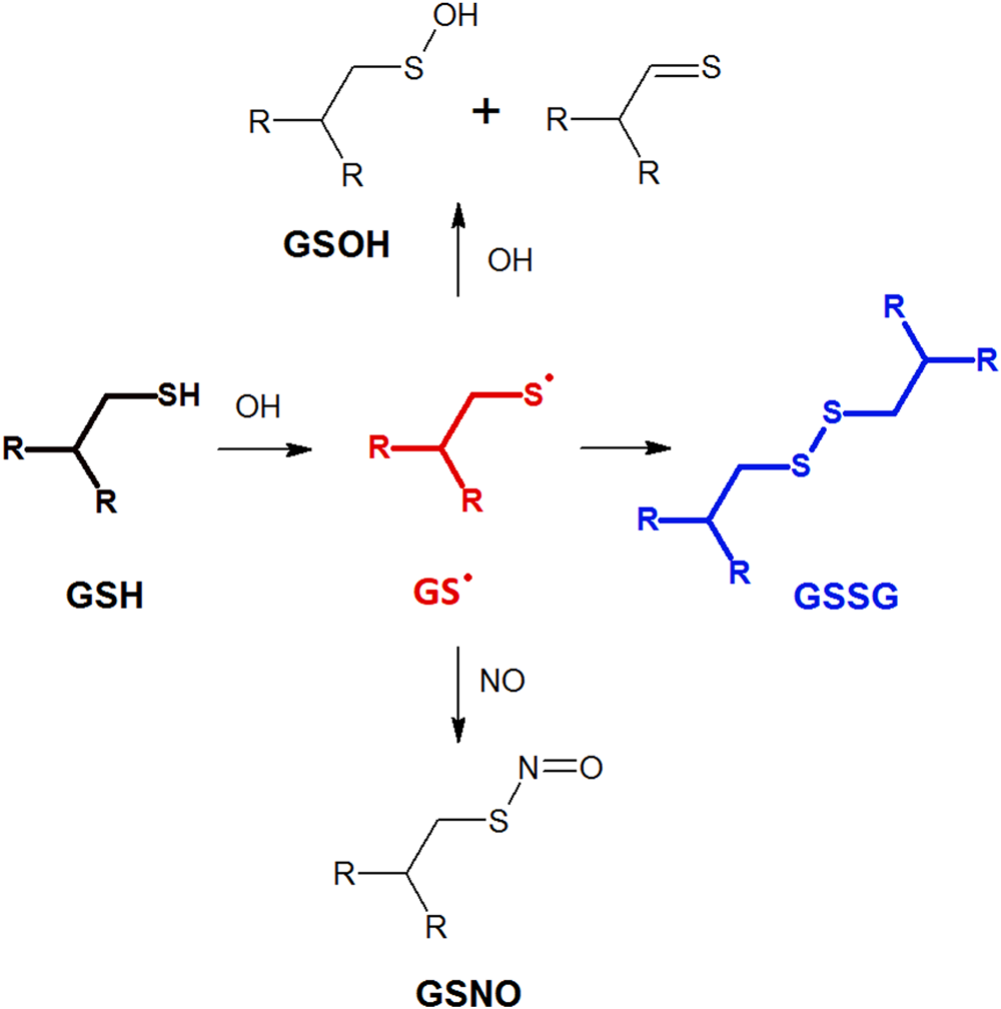
Summary of the reactions between GSH and various reactive species as observed during the reactive MD simulations. The initial GSH structure is indicated using a bold black structure. The resulting GS•, after H-abstraction by OH, is indicated in red. Interactions with reactive species that did not lead to chemical reactions are not depicted.

**Figure 7:**
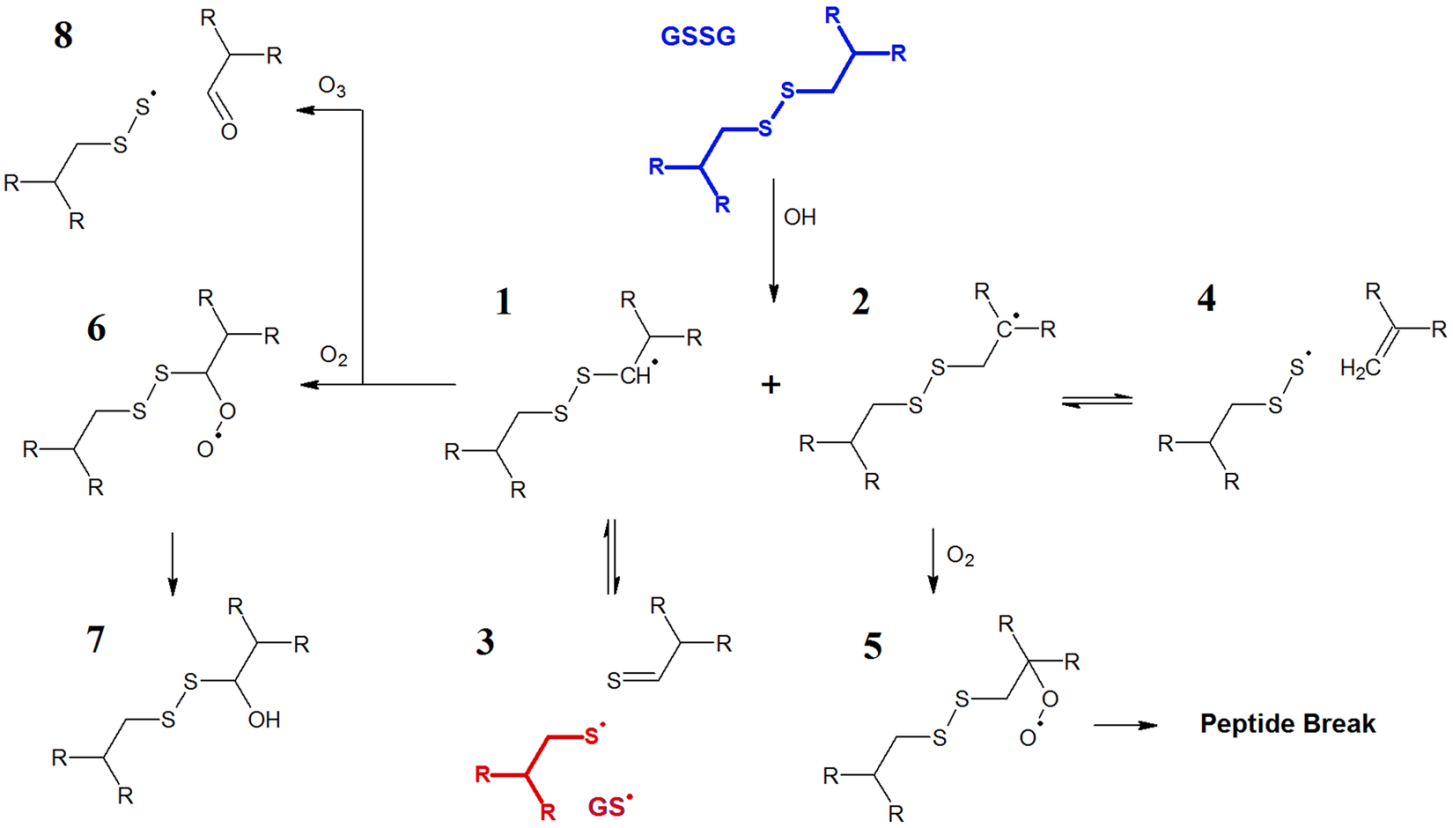
Summary of the reactions between GSSG and various reactive species as observed during the reactive MD simulations. The initial GSSG structure is indicated in blue. The GS• indicated in red is able to react further as depicted in Fig. 6 (online figure in colour). All observed oxidation products are numbered for the sake of clarity. Interactions with reactive species that did not lead to chemical reactions are not depicted The formation of product 7 and the peptide break were not simulated as explained in the text.

Density functional tight-binding (DFTB) simulations were performed to obtain insight in the molecular-scale processes in order to shed light on the observed changes in the chemical structure of both GSH and GSSG. The results are depicted in Figs 6 and 7 (for GSH and GSSG, respectively). It should be noted that only interactions with reactive species that resulted in a chemical reaction are depicted in both figures. Taking this into account, the simulations indicated that oxidation processes were initiated by OH radicals. Interactions with reactive species that did not lead to chemical reactions are not depicted. Other species, such as H_2_O_2_ or NO, only showed weak (attractive or repulsive) interactions with the introduced biomolecule as no chemical reactions were observed within the 10 ps simulated. However, it should be noted that reactions with these species cannot be excluded by the simulations and might still occur after significantly longer timescales.

Interactions between GSH and OH resulted in the abstraction of the thiol H-atom, leading to the formation of a water molecule and a glutathione radical (GS•), located on the cysteine. Once the GS• was formed, reactions with multiple other species could occur. Introducing NO to the resulting radical system led to an addition reaction on the S-atom towards the formation of GSNO. However, when a second OH radical was introduced to the system, either an addition, similar as in the case of NO, or a second H-abstraction, from the C-atom adjacent to the sulphur, could occur. This results in either glutathione sulphenic acid (GSOH) or a S = C double bond, respectively. Interestingly, disulphide bond formation has been observed during the simulations when the GS• radical (as a result of the H-abstraction of GSH) came in contact with a GSH molecule, leading to the formation of GSSG (depicted in blue). While the full GSH molecule has been simulated in solution, chemical modifications were only encountered on the cysteine moieties of this biomolecule, in particular at the thiol group.

Unlike GSH, the initiation sites for oxidation of GSSG were the C-atoms of cysteine (see Fig. 7). Here, OH radicals interacted with GSSG through H-abstraction reactions on either of the two C-atoms of cysteine, leading to either product 1 or product 2 in Fig. 7 (product 1 has been encountered in 40 % of the cases while product 2 has only been encountered in 20 % of the cases). As a consequence, an equilibrium was found between the radical and a broken structure, in both cases. Indeed, it has been observed that the disulphide bond breaks after the abstraction of the H-atom found in the side group of cysteine (see product 1 and 3). The disulphide bond break resulted in the formation of a GS• radical which is prone to further oxidation, as observed in Fig. 7. In case the H-abstraction occurred on the C-alpha position (product 2), the S-C bond was often observed to break leading to an formation of C = C double bonds (product 4). This bond break was, again, found to be in equilibrium with product 2. Interesting is the fact that both GSSG-radicals (1–2) were able to react with molecular oxygen leading to the formation of peroxides (ROO•) as seen in products 5 and 6. It is known from literature that ROO• species are able to react further towards the formation of alcohol and carboxyl groups on the structure^38^. These last steps were not simulated as this would require more complex systems, as well as longer timescales, both beyond the limitations of the used computational method (DFTB). However, using this knowledge we expect the formation of a hydroxyl group adjacent to the disulphide bond (as seen in product 7) as well as a peptide break after the oxidation of peroxide 5. Finally, O_3_ was observed to be able to react with product 1 in rare cases (8 % of simulations). This led to the oxidation of the C-atom adjacent to the disulphide bond resulting in the formation of an aldehyde and the dissociation of the S-C bond (products 8).

## Discussion

Dielectric barrier discharges in ambient air produce reactive oxygen and nitrogen species (RONS)^9^ inducing numerous oxidative modifications in a variety of biomolecules^24,31^. The GSH/GSSG pair serves as a reaction partner for oxidants and redox system for detoxification in organisms and is therefore an interesting target molecule for plasma treatment. Here, we used a combination of physico-chemical techniques (Raman spectroscopy and mass spectrometry) and reactive MD simulations of reactive species to investigate the impact of DBD treatment on GSH and GSSG. Experimentally, the combination of Raman spectroscopy and MS enabled us to observe several modifications, which were also found by MD simulation. While simulations covered only a very short time frame, they allow a view of possible reaction intermediates to further understand the underlying reaction pathways. In contrast, wet-lab experiments can only determine the stable products and/or intermediates making this combined approach ideally suited to observe the complete reaction chain.

The characteristic signals of GSH and GSSG, namely the S-H and the S-S stretching bands respectively, disappeared gradually implying modifications of the sulphur moiety. Simultaneously, the appearance of the S = O stretching mode indicated the formation of GSO_3_H. Analysis of the samples by MS revealed that all of the three possible species of S-oxidised GSH, namely GSOH, GSO_2_H, and GSO_3_H, are produced with GSO_3_H being the most prominent signal after 5 min of treatment, indicating that the thrice-oxidised thiol is the final oxidation product. Another interesting modification observed for both GSH and GSSG is the formation of GSNO that still occurred even after 20 min of treatment. Raman spectroscopy verified the existence of this compound by the appearance of the S-N stretching and the S-N = O bending modes at 522 cm^−1^ and 888 cm^−1^, respectively. This was confirmed by MS with the detection of a compound with a mass-to-charge ratio of 337.08 that correlated to GSNO, although the corresponding signal was much less intense. While the GSNO signal always showed only a small number of counts, it was present in all plasma-treated samples, indicating a stable generation of GSNO by DBD treatment. As the intensities the of the MS signals could not easily be quantitatively correlated, it is challenging to discern if under the present conditions only little GSNO is generated or if GSNO is just ionized sub-optimally.

In the MD simulations, GSNO was formed upon NO addition to GS• In organisms, GSNO acts as one possible NO-donor for protein S-nitrosation which is thought to be related to several pathological conditions, such as neurodegenerational diseases and inflammatory responses and due to its diverse capabilities several studies have already investigated its therapeutic applicability (reviewed, *e.g*., in^39^). In addition, after treatment of GSH, some signals could be annotated as GSSG carrying additional oxygen modifications. Since the sulphur seems to be the most reactive moiety of the molecule, we postulate that the additional oxygen atoms are bound to the sulphur and that this might affect the stability of the S-S bond. However, further in-depth studies will be required. While Raman spectra allowed no clear observation of the disulphide cleavage due to the characteristic peak of ν(S-S) overlapping strongly with the ν(S-N) peak, MS enabled for a clear distinction between the two species. Here, it also became apparent that GSSG acts more like a reaction intermediate when GSH is exposed to the discharge. While GSSG was the most intense signal after 1 min of DBD treatment, the relative signal intensities decreased again for treatment times longer than 3 min. For 5 min of treatment, GSSG could still be found but with strongly reduced counts. In summary, these observations indicate that GSSG acts more as an reaction intermediate. These results were supported by MD simulations, showing that GSSG could indeed be broken down again into two short-lived molecules (reaction 3 in Fig. 7), which can be further modified. Taken together, these findings demonstrate that while GSSG is indeed formed during plasma treatment, extensive treatment can cause an over-oxidation of the disulphide bond resulting in the two end products GSNO and GSO_3_H with a strong preference for the latter. There are three potential pathways for the formation of GSSG from GSH. First, according to the simulations, GS• can react with a GSH molecule. Second, GSNO can react with GS• to GSSG releasing NO^40^. Third, the oxidation to GSOH is reversible meaning that GSOH can react with GSH producing GSSG^41^. The subsequent breakage of the disulphide bond by reactive oxygen species using a DBD was also observed in several proteins as described in^24^. A possible explanation is offered by Stinson and Xia who describe the cleavage of disulphide bonds by reactive species^42^ and the saturation of the resulting sulphur residues seems likely. The MD simulations supported this as the disulphide bond was cleaved as a consequence of a subsequent OH addition to the resulting glutathionyl radical (GS•). Besides the formation of GSSG, the simulations revealed GSOH as oxidation product for GSH after H abstraction, and GSOH can be further oxidised to GSO_2_H and GSO_3_H, while the latter might also be produced via intermediate glutathione peroxide formation upon superoxide addition. These consecutive reactions are also described in^41^. However, both higher oxidation states GSO_2_H and GSO_3_H are considered to be irreversible in organisms^43^ and introduction of these species into the cellular redox system should be performed with care. Furthermore, the Raman spectra showed the disappearance of the peak at 686 cm^−1^ for both GSH and GSSG that was assigned to ν (C-S), indicating the cleavage of the respective bond. However the Raman data did not reveal any evidence to the products. In the mass spectrum, we found species of m = 387.04 Da that correspond to the persulphide GSSO_3_H. In addition, the corresponding GSH carrying a hydroxyl group instead of its thiol group was observed, although both products were only found in low intensities. Simulated reactions with persulphide products showed either an alkene or aldehyde group. However, taking into account the short time spans simulated, it can be expected that alkenes can be further modified, leading to the formation of the observed alcohols after OH-addition^44^. Indeed, as only a few picoseconds could be calculated within a reasonable amount of time, longer-term processes cannot presently be investigated computationally. Accordingly, the MD simulation suggested C-S bond breaking, producing GSS• and an aldehyde or alkene species as counter fragments, respectively (see products 4 and 8 in Fig. 7). These fragments might undergo further reactions as we did not find any experimental evidence for those. Furthermore, the MD simulation indicates that C-S cleavage in GSH is not favourable but can occur via intermediate GSSG. However, it should be noted that other important plasma-generated species such as singlet oxygen and peroxynitrate were not considered by the simulations as they could not be described accurately by the DFTB force field. As both species play an active role in the plasma-induced oxidation of biological tissues, it is expected that their presence can have an effect on the chemical modifications of GSH as observed experimentally.

During plasma treatment the aqueous solution became strongly acidic. However, this does not resemble the conditions of plasma treatments of organisms. Heuer *et al*. treated a reconstructed epidermis model and observed a reduction of pH to about 2.6^45^, though Helmke *et al*. demonstrated that plasma-induced acidification is reversible after about 2 h^46^. Furthermore, the effect of pH was not considered during the simulations given the small dimensions of the calculated system. Therefore, the experiment was repeated in phosphate buffered solution in order to stabilize the pH. In these experiments, the same major oxidation products as for the unbuffered samples were detected. Nevertheless, we observed some differences in the Raman spectra. First, for GSH the ν(S-H) band disappeared much faster in the phosphate buffered samples than in the unbuffered. For GSSG the decay of the ν(S-S) band and the increase of the ν(S-N) signal could not be clearly recognised due to the overlap of the peak with a phosphate band. Second, for both GSH and GSSG the change in intensity from the ν (COO^-^) peak at 1415 cm^−1^ to the ν (COO-H) peak at 1745 cm^−1^ did not occur. This can simply be explained by the suppression of protonation of the carboxylic group that occurs in the unbuffered samples due to the pH drop. Besides, just like for the unbuffered samples we observed the accumulation of GSO_3_H as final oxidation product, as well as the formation of GSNO and the decay of the ν (C-S) band.

Taken together, the present work shows the influence of plasma treatment on GSH and GSSG levels. In order to investigate the chemical modifications of GSH and GSSG we used Raman spectroscopy and MS measurements supported by MD simulations. The time-dependent measurements of DBD influence on GSH revealed the loss of free thiols after plasma treatment within 5 min. Analysis of Raman spectra indicated the oxidation of the sulphur atoms from thiols to S-O, S = O, S-S and S-N = O groups, yielding GSO_3_H, GSSG and GSNO. GSO_3_H seems to be one final product which accumulated with longer treatment times and also GSNO seems to be a rather stable oxidation product. Our findings suggest that these two compounds are formed via two different reaction pathways, one involving reactive oxygen species leading to GSO_3_H, while the other is based on reactive nitrogen species generating GSNO. In contrast, GSSG appears to be an intermediate product that was formed initially but then got further oxidised. MS measurements confirmed oxidation of GSH showing the formation of GSO_3_H, GSNO and further of GSSG as presumed products after short treatment times. However, GSSG was the most prevailing molecule only after 1 min of DBD treatment. After extended treatment time, GSO_3_H appeared to be the most dominant molecule, and still significant amounts of GSNO could be detected. Further, it turned out that GSSG behaved more likely as an intermediate. These results and conclusions were fully supported by MD simulations. Based on our results, we anticipate that for medical applications short and moderate DBD treatment is preferred over the extended time, as previously also suggested by Kisch *et al*.^47^. This is consistent with the experiences from clinical trials where treatment times range from 45 s^27^ to 90 s^28^, and repetitive short treatment times were favoured^47^. Moreover, these results will have to be transferred to future *in vivo* biological investigations on human cell lines, such as keratinocytes.

## Materials and Methods

### Plasma setup

**Figure 8:**
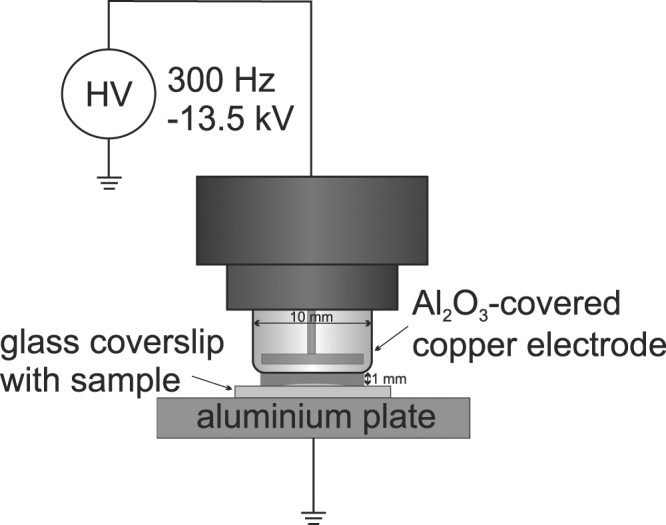
Overview of the employed plasma source. Samples were treated on the glass slide, resulting in a homogeneous discharge in the 1mm gap.

The DBD used in this study consists of a cylindrical copper electrode covered with aluminium oxide (Al_2_O_3_) (see Fig. 8). The electrode with a diameter of 10mm is driven by a pulsed power supply using −13.5 kV at 300 Hz. Samples placed were on a grounded aluminium plate with the distance between samples and driven electrode kept constant at 1 mm. Ambient air was used as process gas. The used source is described in more detail in Bibinov *et al*.^48^. Investigations regarding the production of reactive species of this specific source are available in^9^. In short, the source is capable of generating a complex cocktail of various reactive oxygen and nitrogen species, such as hydroxyl radicals and nitric oxide.

### Biological sample preparation

L-glutathione in oxidised (GSSG) and reduced (GSH) state was purchased from Sigma Aldrich (#G4376 and #G4251, respectively) and dissolved in distilled water with a concentration of 10 mg/ml. For the pH-dependent experiments, the powder was dissolved in sodium phosphate buffer (0.15 M, pH 7). 10 μl were placed on a glass cover slip and treated with the DBD for 1 to 20 min. After treatment, samples were filled into reagent tubes and evaporated liquid replenished with *A. dest*. As controls, another sample was prepared equally, omitting the plasma treatment. Control samples were placed in ambient conditions as the sample treated for the longest time. Prior to the Raman measurement, samples were dried and kept in a desiccator. Samples used for Ellman’s assay or MS were kept at −80 °C prior to measurement.

### Ellman’s assay

Free glutathione after plasma treatment was observed using the glutathione detection kit commercially available from Enzo Life Science (#ADI-900-160) without glutathione reductase resulting in a typical Ellman’s end point detection of free thiols, which react stoichiometrically with 5,5′-dithiobis-(2-nitrobenzoic acid) (DTNB) by cleaving the disulphide bond yielding 2-nitro-5-thiobenzoate (TNB^-^). This is deprotonated forming a yellow compound that can be photometrically quantified at 414 nm directly revealing the concentration of free thiols.

### Raman spectroscopy

Raman measurements were performed on a Raman microscope WITec alpha 300 RAS (WITec) equipped with a frequency-doubled Nd:YAG laser with a wavelength of 532 nm operating at 17 mW. The laser radiation was coupled into the microscope by a single mode optical fibre and focused on the sample by a 100x objective lens (Olympus MPLFLN100X, NA = 0.9). The scattered light was collected by the same objective and detected by the spectrometer unit (UHTS300) consisting of a diffraction grating (600 grooves per mm) and a back-illuminated electron multiplying charge-coupled device (1600 × 200 pixels, cooled to −60 ºC). The spectra were acquired in the spectral range of 0–3700 cm^−1^ using WITec control 1.60. Per sample, 10 spectra were collected at different positions. For every position ten spectra were measured with an integration time of 2 s for each spectrum, averaged automatically by the software. All experiments were performed in triplicates.

### Data processing

For further data processing the spectra were transferred to Matlab R2014b. Background correction was performed using a linear correction (samples in water) or the *msbackadj* algorithm (buffered samples). Afterwards, the spectra in water were normalized to the CH stretching peak at around 3000 cm^−1^. Due to strong phosphate peaks of varying intensity for the samples in phosphate buffer, difference spectra were generated. Sample and buffer-only spectra were normalized to a specific phosphate peak at 500 cm^−1^ and subtracted from each other and resulting difference spectra were normalized to the CH stretching peak.

### Mass spectrometry

Electron spray ionization (ESI) mass spectra were obtained on an Esquire 6000 mass spectrometer (Bruker). Full mass spectra of the investigated GSH and GSSG were acquired in both negative-ion and positive-ion mode with the spectrometer equipped with an ion-trap analyser. Two samples of 10 μl treated for the same time were pooled and diluted tenfold with acetonitrile-water (50:50) for 200 μl with a final concentration of 1 mg/ml. Instrumental parameters were tuned for each sample with GSH or GSSG. The capillary voltage was set in a range of −22 to 25 V, the spray voltage was between 3.00 and 4.50 kV, and a capillary temperature of 180 ºC was employed. The mass scan range was from m/z 50 to 2000 amu, for 20 s scan time. Spectra were acquired using a direct infusion setup with a flow rate of 5 μl/min with a cone voltage of 20 kV. To determine occurring in-source fragments, which increase the sample complexity without yielding significant additional information, MS/MS spectra of both GSH and GSSG were acquired using the same conditions with a collision energy ramp between 2.00 and 4.00 eV. Spectra were deconvoluted and a background of ten times noise (500 counts in positive and 5 counts in negative mode) was subtracted before peak annotation. All experiments were performed in duplicates.

### MD simulations

The chemical modifications of GSH and GSSG, as a result of the interactions with various reactive species (*i.e*. OH, O_2_, O_3_, H_2_O_2_, and NO) were investigated using reactive MD simulations. MD simulations were performed using the density-functional based tight-binding (DFTB) method. The third order DFTB (complemented with the 3ob parameter set) was used utilizing the third series expansion of the total Kohn-Sham energy, for the calculation of the forces between every pair of particles. This resulted in a self-consistent field algorithm taking into account the charges and electron distributions of the atoms. This allowed an accurately description of highly charged biomolecules, binding energies and proton affinities for systems up to 1000 atoms. More information can be found in the work of Elstner *et al*.^49^ and Gaus *et al*.^50^. Prior to the MD impact simulations, the GSH or GSSG molecule was introduced in a simulated cube with 25 Å edge length filled with equilibrated water molecules ensuring a density of approximately 1 g/ml around the biomolecule at all times. The system was further equilibrated at room temperature for 10 ps using a canonical ensemble (NVT where the temperature and volume was kept constant) to eliminate any stresses from introducing the biomolecule into water. A single reactive species was introduced per simulation by replacing a water molecule with the corresponding reactive species. All impact simulations were performed at room temperature for 10 ps using a Berendsen thermostat with a coupling constant of 100 fs and using periodic boundary conditions. Finally, all simulations were performed using a time step for integration of 0.25 fs. Each interaction has been simulated independently for 25 times.

### Data availability

The datasets generated and analysed during the current study are available from the corresponding author on reasonable request.

## Electronic supplementary material

Supplementary Table S1
